# Holding Complexity: Psychological Flexibility and Cultural Identity in a Superdiverse Urban Context

**DOI:** 10.3390/bs16020195

**Published:** 2026-01-29

**Authors:** Anna Medvetskaya, Lisa Stora, Marina Doucerain

**Affiliations:** 1 Département de Psychologie, Université du Québec à Montréal, Montréal, QC H3C 3P8, Canada; medvetskaya.anna@courrier.uqam.ca; 2 Psychology Department, University of Guelph, Guelph, ON N1G 2W1, Canada; lstora@uoguelph.ca

**Keywords:** multicultural identity, superdiversity, place identity, psychological flexibility, latent class analysis, acculturation

## Abstract

Acculturation, traditionally described as the adjustment to new cultural realities, faces major conceptual challenges in superdiverse societies where defining “heritage” and “mainstream” cultures is increasingly problematic. This study proposes expanding cultural identity frameworks to include belonging to one’s living place, emphasizing the ecological significance of place–people relationships in identity formation. We apply this approach to a highly diverse urban context, examining how individuals from both majority and minority backgrounds construct multicultural identities across local, regional, and national levels. A community sample (N = 276) completed measures of cultural identification, psychological flexibility, discrimination, and well-being. Latent class regression revealed four distinct identity profiles: High contextual identification, Low contextual identification, Quebec focus and Montreal focus. Contrary to expectations, the most prevalent profile reflected strong local attachment rather than robust endorsement of multiple cultural levels. Psychological flexibility predicted membership in the High contextual identification profile above and beyond well-being and ambient discrimination, while minority status and perceived discrimination were associated with locally focused profile. These findings underscore the importance of place identity and psychological flexibility in acculturation processes, offering a broader and more context-sensitive account of multicultural belonging in superdiverse societies.

## 1. Introduction

Because of active international mobility, more cultural groups are in contact today than ever (United Nations, 2020), exposing both migrating and non-migrating populations to multiple cultural influences. These global movements have given rise to *superdiverse* societies (Vertovec, 2007), in which cultural heterogeneity extends well beyond ethnicity to encompass variations in language, migration histories, and socio-economic backgrounds. Importantly, this perspective reflects a broader shift across disciplines toward understanding culture and identity as constituted through multiple, overlapping reference points rather than as anchored solely in national belonging. In such contexts, individuals are continuously required to negotiate their positioning across several cultural frameworks—a process that reshapes how cultural identity is experienced and expressed. Traditionally, such processes have been conceptualized under the rubric of acculturation, defined as cultural and psychological change following contact between cultural groups, often in migration contexts (Berry, 2005). In contemporary superdiverse settings, however, acculturation may also involve navigating multiple, coexisting cultural reference systems within a single social environment (Schwartz et al., 2006), reflecting how people construct and navigate their (multi)cultural selves in environments of complex, layered diversity that current research has yet to fully capture.

Cultural diversity is a hallmark of the 21st century (Miller & Collette, 2019) and acculturation concerns both migrating and non-migrating populations (Kunst et al., 2021). Yet, cross-cultural psychological research on cultural identification still largely relies on traditional immigrant-focused bidimensional models centred around two primary cultural reference points: a mainstream culture (i.e., the dominant cultural stream in a migrant’s new country) and a heritage culture (i.e., the dominant cultural stream in a migrant’s country of origin). While such models are bidimensional and explicitly allow for biculturality and marginalization (Berry, 2003, 2005), they remain structurally limited to the negotiation of two cultural streams. This two-stream structure, however, is limited in addressing multicultural identity as a “condition of having strong attachments with and loyalties toward different cultures” (Nguyen & Benet-Martínez, 2010, p. 4), particularly in contexts where culturally meaningful identifications extend beyond a single heritage–mainstream pairing. Indeed, “different cultures” often refers to more than two cultures.

Theoretically, multicultural identity has typically been addressed as an extension of the traditional mainstream-heritage bicultural identity. Such unquestioned extension is problematic for at least two reasons. First, defining a mainstream culture in superdiverse settings (Kunst et al., 2021; Zagefka et al., 2023) is difficult at best. Second, traditional models tend to ignore local contextual influences of superdiverse environments on multicultural identity. From an ecological perspective on the self-concept, place-people relationships are crucial in identity construction processes since places (i) are environments in which social/cultural processes unfold and (ii) symbolize these social/cultural processes (Hormuth, 1990; Lalli, 1988). Yet, acculturation research usually overlooks the role of place as a source of cultural meaning. Accordingly, comparing identification with the city, province, and nation allows assessing how cultural identifications are structured across contextual levels of symbolic belonging in superdiverse settings. This work targets these issues by investigating identification patterns with three cultural streams (local, provincial, national) in a superdiverse setting and by including city identification as a distinct component of both majority and minority individuals’ multicultural identity. Importantly, in the present study, *culture* does not refer to bounded ethnic, religious, or heritage groups, but to institutionally and symbolically constituted cultural frames of belonging (e.g., local, regional, national) that structure meaning, norms, and recognition within the cultural mainstream. Further, distinctiveness refers to the empirical differentiation of identification dimensions within the self-concept, rather than the motivational need for intergroup distinctiveness as defined in social identity theory (Brewer, 2012). These dimensions refer to differentiated facets of identification within the self-concept, not to cultures as bounded or homogeneous entities.

Another point to consider is that constructing a multicultural identity can be challenging. Navigating a culturally diverse environment with differing expectations, norms and values and juggling one’s loyalties to cultural groups (Hong et al., 2007) requires a certain degree of psychological flexibility. Thus, we also assess psychological flexibility as a correlate of multicultural identification patterns in the multicultural context of Montreal, a superdiverse metropolis in the province of Quebec, Canada.

## 2. Multicultural Identity: Some Gaps

Despite increasing recognition that many individuals hold multiple cultural allegiances, psychological theories of multicultural identity remain fragmented (Nguyen & Benet-Martínez, 2010). Multicultural identity has been variously conceptualized: as the synthesis of diverse cultural perspectives into novel cognitive styles and coping strategies (Ramirez, 1995); as an ongoing process of meaning-making across contexts (Greenholtz & Kim, 2009; Lapresta & Huguet, 2008); or as a flexible repertoire of shifting identifications depending on situational demands (Moore & Barker, 2012). Other perspectives emphasize the complex experience of balancing one’s sense of uniqueness and detachment from any particular culture with the commitment to collaborate with one’s local communities (Sparrow, 2013). These approaches have primarily conceptualized multicultural identity as an adaptive process within individuals, often centered on negotiating between heritage and host cultural frameworks. Empirical investigations of multicultural identity, therefore, are usually limited to combinations of two cultural identifications, with only a few exceptions (e.g., Carpentier & de la Sablonnière, 2013; Downie et al., 2004).

Berry’s (2003, 2005) widely adopted acculturation model—where acculturation refers to “the process of cultural and psychological change that results from contact between two or more cultural groups” (Berry, 2005, p. 100)—is a case in point. In superdiverse settings, such contact is not limited to interactions between distinct populations, but also occurs through individuals’ everyday navigation of overlapping institutional, linguistic, and political cultural frames within the same social space. While theoretically acknowledging the potential of acculturative processes to foster multicultural identification (Berry, 2003), in practice, this model delineates acculturation patterns solely along mainstream and heritage dimensions. It proposes four acculturation strategies: integration (combining heritage and dominant cultures), assimilation (adopting the dominant culture while abandoning heritage traditions), separation (preserving heritage culture while avoiding the dominant one), and marginalization (rejecting both). This model fails to explain integration in cases that involve more than two cultural identifications, a limitation that has already drawn criticism (e.g., Benet-Martínez & Haritatos, 2005; LaFromboise et al., 1993). This being said, even models developed specifically to account for multiple cultural identifications tend to consider only two in their explanations (e.g., Amiot et al., 2007; Roccas & Brewer, 2002). The present study does not seek to extend acculturation models to additional cultural domains, nor to examine adaptation between heritage and host cultures. Instead, it addresses a distinct question: Whether identification with the cultural mainstream itself becomes context-specific and differentiated across local, regional, and national levels in superdiverse settings.

## 3. Multicultural Identity in Superdiverse Settings: Further Challenges

Building on critiques of the mainstream–heritage dichotomy, scholars increasingly note that the very notion of a unified “mainstream” culture is eroding in superdiverse societies with strong migration currents such as Australia, Canada, Western Europe, or Singapore (Doucerain et al., 2024; Nguyen & Benet-Martínez, 2010). In such contexts, the cultural mainstream itself may no longer function as a singular reference point, but rather as a set of overlapping and contested identity streams anchored in different places (e.g., city, region, nation), each representing a unique sociopolitical and historical context. In cities marked by migratory flux, linguistic plurality, and sociopolitical contestation, cultural belonging cannot be easily mapped onto singular national narratives. Montreal offers a particularly compelling context to study these dynamics. This is not only because of its high level of demographic diversity, but because the Canadian identity landscape itself is internally differentiated across local, regional, and national levels. In this context, Montreal, Quebec, and Canada function as distinct cultural–institutional frames of belonging within the mainstream, rather than merely as nested geographic units. As the largest city in Quebec—a province whose cultural distinctiveness is fiercely defended through language and policy—Montreal exemplifies how regional, national, and local identity claims may coexist, overlap, and sometimes conflict.

Quebec’s interculturalism policy emphasizes interculturalism, rooted in French language dominance and cultural traditions, which symbolize a unique Quebec identity (Mathieu, 2022). Unlike Canada’s broader multicultural framework, which highlights cultural plurality as a cornerstone of national identity (Ryan, 2010), Quebec asserts itself as a distinct nation, even using its cultural uniqueness to bolster claims for independence (Paquin, 2018). Canada has embedded multiculturalism in its constitution (Brosseau & Dewing, 2018), emphasizing the importance of respecting and ensuring equal civic participation for all cultural groups. In contrast, Quebec’s interculturalism emphasizes the integration of diverse cultures into a cohesive society that prioritizes the French language and Quebecois culture (Proulx-Chénard, 2021). The consequences of these differing policies are evident in societal dynamics. Multiculturalism in Canada has been criticized for potentially leading to cultural fragmentation, as it may encourage the formation of ethnic enclaves with limited interaction between groups. Conversely, Quebec’s interculturalism aims to mitigate such fragmentation by promoting intercultural dialogue and integration into the francophone majority. However, this policy has faced criticism for potentially imposing assimilation pressures on ethnic and cultural minority groups, as it places a strong emphasis on adopting the French language and aligning with Quebecois cultural norms (Wences, 2016). These dual dynamics of a Quebec-specific identity and Canada’s multicultural ethos complicate notions of “mainstream” culture, especially in superdiverse urban centers. Consequently, “Québécois” and “Canadian” may both be claimed as “mainstream” identities in Quebec. As a result, perceptions and endorsements of the mainstream identity may vary qualitatively and quantitatively across the country (Berry & Hou, 2021).

Further, multicultural identity development does not occur in isolation from dominant group dynamics. Exposure to cultural diversity reshapes not only minority experiences but also majority group identifications (Kunst et al., 2021; Zagefka et al., 2023). In cosmopolitan environments like Montreal, identification with Quebec among individuals from longstanding Quebecois backgrounds may become infused with multicultural elements. Openness toward other cultures can vary widely from minimal acknowledgment to active engagement with diverse cultural practices, and, at its fullest, may even expand the symbolic boundaries of what is considered “mainstream” identity (Doucerain et al., 2024). Importantly, the construction of multicultural selves among minoritized individuals is shaped not only by personal openness but also by the dominant group’s willingness to recognize and include them within collective narratives of belonging (Alba & Duyvendak, 2020). Ethnic, racial, religious, and linguistic identities are therefore not treated as alternative outcomes in the present study, but as background conditions that structure access to, and recognition within, local, regional, and national mainstream identities. Thus, while demographic diversity certainly contributes to Montreal’s superdiversity, the present study focuses specifically on how the cultural mainstream itself becomes pluralized and contextually differentiated in such settings.

## 4. Context Influences: Place as a Cultural Identity Resource

The formation of multicultural identities is deeply situated in the relational and spatial dynamics of everyday life. Interactionist models of identity emphasize that cultural belonging emerges through dynamic exchanges between individuals and their environments, shaped by symbolic meanings, institutional structures, and lived experiences of inclusion or exclusion (Giddens, 1984; Goffman, 1959). In superdiverse cities, the local context itself can become a primary cultural resource, offering a “culture of multicultural possibilities” (i.e., a space in which contacts with multiple cultures are possible), more immediate and emotionally resonant than abstract national identifications.

From an ecological perspective, place is a formative force for identity. Proshansky’s (1978) model of place-identity highlights how environments embed “complex patterns of conscious and unconscious ideas, feelings, and values” into the self (p. 155). Moreover, place-people relationships are not passive: People perceive and shape the identity of the place, which is rooted in physical, symbolic, institutional, and other characteristics (Peng et al., 2020). In turn, the specific identity of a place affects identification patterns of its inhabitants (Licari, 2011; Twigger-Ross et al., 2003).

The extent to which place identity could be seen as a cultural identity depends on the extent to which place can be seen as “culture-full.” Scholars in social and urban studies have long argued that place itself is a cultural phenomenon, whenever it is created by humans (Lefebvre, 1974/1991; Low, 2000, 2009; Richardson, 1982). This is especially true in the age of globalization, when large urban centres become *global cities* (characterized by high economic power, increased flow of capital and information, and cultural diversity) that act as “cultural hubs” where rapid and dynamic cultural exchanges take place (Pooch, 2016). These cultural exchanges are a prerequisite for acculturative changes in behaviours, values, and identities (Schwartz et al., 2010). Despite the potential of a city to shape cultural identity, ecological perspectives on the self have not received deserved attention within cross-cultural psychology (Jurcik et al., 2013) and social psychology more broadly (Lewicka, 2011). In cities like Montreal—where regional, national, and global currents collide—cultural hybridity is not simply interpersonal but materially and symbolically inscribed in everyday life. Urban spaces provide fertile ground for cultural negotiation and symbolic expression (Low, 2000, 2009), fostering constant negotiation and recombination of cultural narratives (Schwartz et al., 2010).

For individuals who are racialized (i.e., socially ascribed minority status through visible markers such as race or ethnicity) or minoritized (i.e., marginalized through sociopolitical hierarchies regardless of visible difference), local place-based identities may offer alternative pathways of belonging when regional or national narratives remain exclusionary. Yet, mainstream psychological models have often neglected place-based identity processes (Jurcik et al., 2013; Lewicka, 2011). This study addresses this gap by examining local (Montreal), regional (Quebec), and national (Canada) identifications as interconnected and contested mainstream cultural identity streams, thereby shifting attention from adaptation between heritage and host cultures to the contextual structuring of belonging within the cultural mainstream itself. Thus, while classic acculturation research focuses on who is in contact with whom, the present study focuses on which cultural frames are made available, salient, and legitimate within the mainstream culture itself.

## 5. Psychological Flexibility as a Resource for Multicultural Identity in Plural Societies

A key question in superdiverse societies is how individuals construct multicultural identities, especially when their self-concept integrates three or more cultural affiliations (Chu et al., 2017; West et al., 2017). This process is cognitively and emotionally demanding, particularly for minoritized individuals navigating dominant cultural narratives (Doucerain, 2019; Pekerti, 2019; Weber et al., 2015). Conceptually, multicultural identity can be understood as a manifestation of *identity complexity*—that is, the psychological organization of multiple, and sometimes intersecting, cultural affiliations within the self (Amiot et al., 2007; Roccas & Brewer, 2002). This complexity does not apply solely to individuals of immigrant background: In superdiverse societies, majority group members are also exposed to diverse cultural frameworks that may diversify and contextualize their own identifications (Doucerain et al., 2024; Kunst et al., 2021). Thus, the notion of multicultural identity inherently implies varying degrees of identity complexity, reflecting how individuals integrate or differentiate multiple cultural reference systems across contexts. Negotiating multicultural identity often involves reconciling cultural contradictions, interpreting ambiguous social cues, and managing psychological effects of discrimination—whether perceived or experienced first-hand—while sustaining a coherent sense of self (Y. Y. Kim, 2015; Miglietta et al., 2023). Such efforts may evoke frustration, alienation, or anxiety (Berry, 1997), underscoring the need for resources that support goal-directed functioning amid emotional discomfort (Tadmor et al., 2009).

One such resource is psychological flexibility—an adaptive capacity to respond to shifting internal and external demands while maintaining commitment to valued goals (Kashdan et al., 2020; Westhoff et al., 2024). We propose that psychological flexibility, though initially conceptualized as a self-regulatory capacity, may also support multicultural identity navigation by helping individuals navigate conflicting cultural expectations and maintain a coherent sense of self in diverse social environments. At its core, psychological flexibility incorporates the concept of self-as-context, which views the self as a stable yet flexible perspective that allows individuals to observe and manage their thoughts, emotions, and identities without excessive attachment to any single one (Hayes et al., 1999). This perspective is particularly relevant for multicultural individuals who engage in cultural frame switching, a process where behaviors, values, and emotional expressions shift depending on the cultural context (Hong et al., 2000). Psychological flexibility also facilitates cultural frame switching, allowing individuals to engage fluidly with different cultural norms without fragmenting their identity (Hong et al., 2000), and fosters emotional integration by encouraging acceptance of cultural dissonance rather than avoidance (Cheng et al., 2014). These capacities reflect resilience mechanisms described in minority stress frameworks, where adaptive regulation and openness buffer psychological distress (Meyer, 2003; Hatzenbuehler, 2009). Research on bisexual individuals suggests that psychological flexibility supports identity integration and a positive self-view despite stigma (Brewster et al., 2013; Hrehorciuc-Caragea & White, 2017). This aligns with the mental resources hypothesis, which proposes that early psychological adjustment facilitates cultural integration (Doucerain et al., 2023).

Flexibility is also relevant for dominant group members. In superdiverse contexts, majority identities themselves may reflect internal complexity—varying across local, provincial, and national reference frames and requiring negotiation of multiple cultural meanings within the self. Psychological flexibility can therefore function as a mechanism that helps majority individuals navigate these overlapping identity layers, integrating diverse cultural frames both internally and in relation to others. At a broader societal level, such flexibility can foster openness to pluralistic identifications, reduce resistance to inclusion, and expand civic narratives around national identification (Medvetskaya et al., 2025). Without this openness, efforts by minoritized individuals to integrate across cultural lines may be constrained by dominant group gatekeeping (Kunst et al., 2021). Importantly, psychological flexibility appears to be malleable and responsive to intervention (Peterson et al., 2024). Enhancing it may promote identity coherence, emotional resilience, and social belonging across sociocultural hierarchies.

While some studies have explored flexibility as an outcome of multicultural engagement (Benet-Martínez et al., 2006; Christmas & Barker, 2014; B. S. K. Kim & Omizo, 2005; Leung & Chiu, 2010), few have investigated it as a predictor of complex multicultural identity construction. Psychological flexibility may thus represent a key psychological resource that supports individuals in navigating identity complexity within superdiverse contexts. However, this process unfolds within broader psychosocial conditions that can either facilitate or constrain adaptive identity integration. From the perspective of minority stress and contextual identity frameworks (Lincoln et al., 2021; Meyer, 2003; Yampolsky et al., 2016), experiences of discrimination, psychological well-being, and developmental factors such as age shape the context in which multicultural identities are negotiated. Discrimination, whether personally experienced or observed toward culturally different others, can undermine a sense of belonging and disrupt identity coherence, thereby testing individuals’ capacity for flexible self-regulation. Further, psychological well-being is strongly associated with psychological flexibility. Including psychological well-being as a covariate enables a more precise assessment of psychological flexibility’s specific effect (Cherry et al., 2021). Age-related variations in flexibility further reflect developmental differences in regulatory and adaptative capacities: Younger adults often show lower flexibility (Okayama et al., 2024; Whitmoyer et al., 2023), although aging-related health issues can also diminish adaptive functioning (Plys et al., 2022). Conceptually, these contextual dimensions do not constitute core psychological mechanisms of identity construction but rather background conditions that modulate its expression. For this reason, in the present study, they are treated as covariates: Contextual factors that may blur the specific contribution of psychological flexibility if left uncontrolled, but that do not themselves form part of the theoretical model under examination.

## 6. The Present Study

This study was conducted in a multicultural sample that included both majority and minority individuals, with two goals. First, using latent class analysis (LCA), we examined multicultural identification patterns along three dimensions: national (Canada), regional (Quebec), and local (Montreal). Capturing these emergent and context-specific identity patterns requires a move beyond variable-centered approaches. Latent class analysis (LCA) offers a person-centered alternative by identifying unobserved groups (latent classes) based on participants’ responses. LCA assigns individuals to these classes with varying probabilities, focusing on observed response patterns rather than relationships among variables. This approach provides a clearer, data-driven picture of the multidimensional nature of cultural identification (Bámaca-Colbert & Gayles, 2010). The choice of cultural identifications was informed by ecological perspectives on the self and by ambiguity surrounding the meaning of “mainstream” identification in Quebec. Rather than including heritage cultural identifications—which would not be applicable to all participants—we focused on dimensions shared across the sample. Second, we tested whether psychological flexibility, conceptualized as a self-regulatory resource supporting complex identity negotiation, would predict class membership, controlling for traditional covariates such as well-being and perceived discrimination.

We conducted the study in Montreal, QC, a superdiverse setting marked by strong regional nationalism, bilingualism (58.5% of the population know both French and English, 59.1% of the city population speak French most often at home, 25.6% speak English most often at home), and a large immigrant population (33.4%) (Statistics Canada, 2022). Quebec’s interculturalism policies have complex effects on identification processes: While they may foster loyalty to Quebec among some groups (Berry & Hou, 2021), they can also alienate immigrants, particularly non-francophones and racialized individuals (Bilodeau & Turgeon, 2023). About one-third of newcomers leave Quebec for other provinces within ten years (Renaud & Goyette, 2002), suggesting that regional belonging is not uniformly attainable. Those who remain often settle in Montreal, whose cosmopolitan identity and bilingualism may offer a more accessible cultural home (Proulx, 2021; Saba, 2024).

In line with place attachment theories, we hypothesized (H1) that Montreal identification will function as a contextually differentiated component of cultural identity, evidenced by (a) higher endorsement relative to regional and national identification and (b) only modest associations with these identifications. Furthermore, LCA profiles will reflect patterns in which local identification is expressed independently from provincial and national identification. Consistent with prior findings favoring integration strategies (Amiot et al., 2018; Berry, 2010), we expected (H2) that a class reflecting strong identification with all three cultural streams would be the most prevalent. Finally, we hypothesized (H3) that higher psychological flexibility would predict membership in the integration-like class, reflecting the cognitive and emotional demands of navigating plural cultural landscapes. To account for contextual factors previously associated with identity development, we controlled for psychological well-being, perceived ambient discrimination, and age. To ensure relevance across minority and majority participants, we measured ambient discrimination—perceptions of unfair treatment toward culturally different others in the broader environment—rather than self-directed discrimination, which may not resonate equally across groups.

## 7. Materials and Methods

### 7.1. The Participants and Procedure

Data were collected during mostly the winter months (November 2021–April 2022), following ethics approval (July 2017). Recruitment took place in public spaces, including streets and metro station entrances, which remain highly frequented across seasons in Montreal. Adults living in Montreal, Quebec, Canada, were recruited through flyers distributed on streets and at the entrances of subway stations. Our flyer distribution scheme covered a large array of the city’s neighbourhoods to make our sample as diverse and representative as possible. Interested individuals accessed the online survey on the Qualtrics platform via link or QR code. To participate, they were required to check a consent box after reading the study information. Those who did not provide consent were redirected to the end of the survey and thanked for their interest. Participants who consented completed a 20-min questionnaire. Inclusion criteria were being at least 18 years old and sufficiently fluent in English or French, operationalized as the ability to read and understand the recruitment flyer and the consent form. This approach is consistent with standard practice in minimal-risk psychological research, where language proficiency is assessed in terms of capacity to provide informed consent and understand study instructions. Individuals who did not meet these criteria were excluded from participation. Participants entered a draw (1/100 chance) to win $100. Ethics approval was obtained from the authors’ institutional board.

Of the 486 individuals who began the survey, only those who completed at least 80% of the items were retained for analysis, resulting in a final sample of 276 participants. In terms of gender identity, 133 identified as women, 136 as men, and 7 selected “prefer not to answer.” Participants ranged in age from 18 to 81 years (*M* = 36.26, *SD* = 14.67). Among them, 62.45% were Canadian-born, and immigrant participants had resided in Canada for an average of 10.57 years (*SD* = 10.63). About 37.54% self-identified as people of color, with 6.49% reporting multiple ethnic backgrounds. Primary languages included French (63.54%), English (7.58%), and other languages (18.41%). Educationally, 57.4% had at least an undergraduate degree. Employment status indicated 59.57% worked full- or part-time, and 23.83% were students. Somewhat more than half of the participants (60.3%) reported having an income of $59,000 or less, 9% preferred not to answer or did not know their income. Although the sample was not probability-based and required sufficient English or French proficiency, several characteristics (e.g., proportion of Canadian-born residents, income range, and educational attainment) showed similarity to census benchmarks for the Montreal metropolitan area (Statistics Canada, 2022). These descriptive comparisons are provided for contextualization rather than as evidence of representativeness, and the findings should be interpreted as reflecting the experiences of a linguistically proficient, urban adult population residing in Montreal rather than Montreal population as a whole.

We operationalized belonging to Quebec’s dominant ethnocultural group as being born in Quebec or elsewhere in Canada, being White, and having at least one parent born in Canada. This operationalization captures individuals who are commonly recognized in public and media discourse as part of the dominant ethnocultural group in Quebec (often referred to as “Québécois de souche” or “Old-stock Quebecers”), including those who migrate internally within Canada but retain full symbolic membership in the majority group. This group included 129 participants (46.57%). The rest of the sample (148 participants or 53.43%) was coded as having a minoritized status. This minoritized group included second-generation immigrants racialized as non-White, based on their struggles to be recognized as Québécois by the dominant group.

### 7.2. Materials

#### 7.2.1. Cultural Identification

We assessed identification with Canada, Quebec, and Montreal using the Inclusion of Ingroup in the Self Scale (IIS; Tropp & Wright, 2001), a pictorial measure of self–ingroup overlap. Participants select one of seven Venn diagram-like images indicating increasing degrees of overlap between “Self” and the target group. This single-item scale has demonstrated construct validity and temporal stability in past research (Tropp & Wright, 2001). Although the Inclusion of Ingroup in the Self Scale is a single-item pictorial measure and internal consistency cannot be computed, we assessed the convergent validity of the IIS using a direct belonging item (“I feel that I fit in in [place]”) for Quebec and Montreal. The IIS showed strong positive correlations with these items (Quebec: *r* = .63, *p* < .001; Montreal: *r* = .57, *p* < .001), supporting its construct validity in these domains. A comparable item was not available for Canada, precluding parallel validation for the Canadian identification measure. However, given the IIS’s consistent theoretical structure across targets and its prior empirical support (Tropp & Wright, 2001), we retained the Canadian version for analysis while noting this as a limitation.

#### 7.2.2. Psychological Flexibility

The Personalized Psychological Flexibility Index (PPFI; Kashdan et al., 2020) measured participants’ adaptive responses to goal pursuit, across avoidance, acceptance, and emotional harnessing subscales on a Likert-type scale from 1 = strongly disagree to 7 = strongly agree. Reliability was acceptable (Cronbach α = .72) in our sample. The PPFI has demonstrated robust psychometric properties in racially and culturally diverse samples, including Chinese medical students (Jiang et al., 2024), Iranian adults (Akbari et al., 2021), and racially diverse U.S. college students (Jo et al., 2023), supporting its cross-cultural validity.

#### 7.2.3. Covariates

Participants rated their current satisfaction with life, psychological health, and perceptions of ambient discrimination with three Likert-type questions: “Overall, how satisfied are you with life as a whole these days?” (1 = extremely dissatisfied to 7 = extremely satisfied); “How would you rate your psychological health?” (1 = poor to 5 = excellent); and “In general, how often do you think people are treated unfairly in Quebec when they have cultures and traditions different from those of the majority?” (1 = never to 5 = always), respectively. We used single-item measures since they significantly reduce respondent burden while still providing valid and reliable assessments comparable to multi-item scales (e.g., Cheung & Lucas, 2014). They are particularly useful in large-scale or diverse studies, offering broad applicability and minimizing common method biases (Ahmad et al., 2014; Hoeppner et al., 2011). Given that participants were recruited at subway stations, the survey was designed to be completed within an average subway ride (approximately 20 min) to ensure higher participation rates and minimize the likelihood of respondents discarding or forgetting the pamphlet.

### 7.3. Analysis

We inspected data for outliers, normality, and missingness. Minor univariate outliers were winsorized; no multivariate outliers were detected. Most variables had minimal missingness (<5%), except Canadian identification (17.39%). Missingness patterns suggested missing-at-random (MAR), confirmed via logistic regression: Higher discrimination and stronger Quebec/Montreal identification were indeed statistically significantly associated with missing values on Canadian identification. Two explanations for this pattern are plausible. People who perceive more ambient discrimination might feel resentment toward the Canadian identity because of a dissonance between its explicit multicultural orientation and the prevalence of discrimination. Alternatively, non-responders might feel stronger identification with the French-aligned Montreal and Quebec identities, thus judging the question about their Canadian identification as irrelevant. Bootstrapping-based imputation (Amelia-II; Honaker et al., 2011) addressed missingness, a recommended method for latent class analysis (Lee & Shi, 2021; Lanza & Cooper, 2016). As a robustness check addressing potential sensitivity to nonresponse on Canadian identification, we repeated the primary latent profile analyses using complete cases only (reported in Supplementary Materials).

We conducted latent class regression using poLCA (Linzer & Lewis, 2011), modeling cultural identifications (Canada, Quebec, Montreal) alongside predictors. Fit was evaluated via BIC, AIC, entropy (>0.70), minimum class size (≥5%), and mean posterior probabilities (>0.70). Table 1 displays goodness-of-fit statistics. Class enumeration proceeded until higher-order models failed to converge. The bootstrap likelihood ratio test (BLRT) remained significant through the 4-class solution (*p* < 0.001); however, a 5-class model was not estimable given the sample size (non-convergence despite extensive random starts), precluding further BLRT comparisons. We therefore selected the 4-class model based on combined criteria: Lowest AIC among converged models (3-class AIC = 2212.53 vs. 4-class AIC = 2190.86) and lower sBIC (3-class sBIC = 2234.55 vs. 4-class sBIC = 2221.43), acceptable classification quality (relative entropy ≈ 0.84; average posterior probability by assigned class = 0.912, 0.893, 0.885, 0.991; overall ≈ 0.90), adequate class sizes (33.3%, 44.6%, 13.8%, 8.3%), and theoretical interpretability (Morgan, 2015; Nylund-Gibson & Choi, 2018). Although the smallest class comprised 8% of participants, we retained it because it reproduces a theoretically expected minority profile and exceeds common mixture-model heuristics recommending avoidance of classes smaller than 5% of the sample (Andruff et al., 2009). Residual item–item associations were examined using poLCAExtra (Caron, 2024), covariance residual checks for the 4-class model. No pairwise residuals reached significance after conditioning on class membership, supporting local independence; accordingly, we did not add direct effects among indicators and retained the 4-class solution on parsimony grounds. Although a two-class model had the optimal BIC/CAIC among converged solutions (e.g., BIC lower at 3-class BIC = 2389.93 vs. 4-class BIC = 2437.04; 3-class CAIC = 2438.93 vs. 4-class CAIC = 2505.04), examination of the two- and three-class models indicated that these solutions primarily reflected differences in overall identification strength rather than qualitatively distinct patterns of cultural belonging. Specifically, lower-order models collapsed local, regional, and national identifications into parallel high–low or high–moderate–low profiles, obscuring the place-based differentiation central to the study’s theoretical framework. In contrast, the four-class solution captured structurally distinct configurations of identification, including profiles in which local identification operated independently of regional and national belonging. Consistent with recommendations to balance statistical criteria with theoretical coherence and construct validity in person-centered modeling (Morgan, 2015; Nylund-Gibson & Choi, 2018), the four-class solution was retained based on converging evidence from AIC and sBIC, significant BLRT results, acceptable classification quality, adequate class sizes, and substantive interpretability. Two- and three-class solutions are reported in the Supplementary Materials for transparency.

To assess the robustness of the latent class structure to modeling assumptions, we conducted a sensitivity analysis treating cultural identification indicators as continuous using Gaussian mixture modeling (mclust). A four-component solution yielded profiles that closely mirrored those obtained in the primary latent class analysis, including (a) a profile characterized by high concurrent identification with local, provincial, and national contexts, (b) a low-identification profile, (c) a subnational profile marked by strong local identification coupled with weak national identification, and (d) provincial identification with moderate identification across contexts. Although information criteria favored a five-component solution, the four-class model reproduced the core substantive configurations central to the study’s theoretical framework, supporting the robustness of the primary findings. Further, the complete-case analyses yielded a substantively similar profile structure, including a differentiated Montreal-focused profile and an integration-like profile, indicating that the primary conclusions were not driven by the imputation of Canadian identification. Our sample size (N = 276) aligns with precedent in similar studies (e.g., Driscoll & Torres, 2022; Bámaca-Colbert & Gayles, 2010), although it precluded separate majority–minority analyses.

## 8. Results

### 8.1. Descriptive Statistics

Table 2 presents descriptive statistics and correlations among variables. Because the cultural identification item is one-item seven-category ordinal measure, we estimated associations using Spearman’s rank correlation, which is recommended for ordinal variables and monotonic associations; when the cultural identification item was paired with a continuous variable, we likewise used Spearman to avoid imposing interval spacing on the ordinal scale (Jamieson, 2004). To assess empirical differentiation across levels of cultural identification, we examined patterns of mean endorsement and rank-based associations (Spearman correlations), complemented by latent-class analysis capturing natural response patterns. Overall, participants identified more strongly with Montreal than with Quebec (*t*(275) = −4.58, *p* < .001) or Canada (*t*(275) = −14.31, *p* < .001), and more strongly with Quebec than with Canada (*t*(275) = −7.85, *p* < .001). As a robustness check, we repeated these comparisons using ordinal nonparametric tests, which yielded identical conclusions (see Supplementary Materials). Montreal identification was positively correlated with both Quebec and Canadian identification, though associations were small to moderate in magnitude. These differences in endorsement, combined with modest correlations among the three identification levels, support the idea that Montreal identification functions as a contextually differentiated component of cultural identity (H1). Quebec identification and Canadian identification were not significantly correlated, indicating that these two levels of identification did not covary systematically in this sample.

Participants showed high psychological flexibility, life satisfaction, and psychological well-being, with no significant differences between majority and minority groups. Scores on ambient discrimination were moderately high and also similar across groups. Psychological flexibility correlated positively with life satisfaction and well-being; ambient discrimination correlated negatively with well-being.

### 8.2. Latent Class Structure

**Figure 1:**
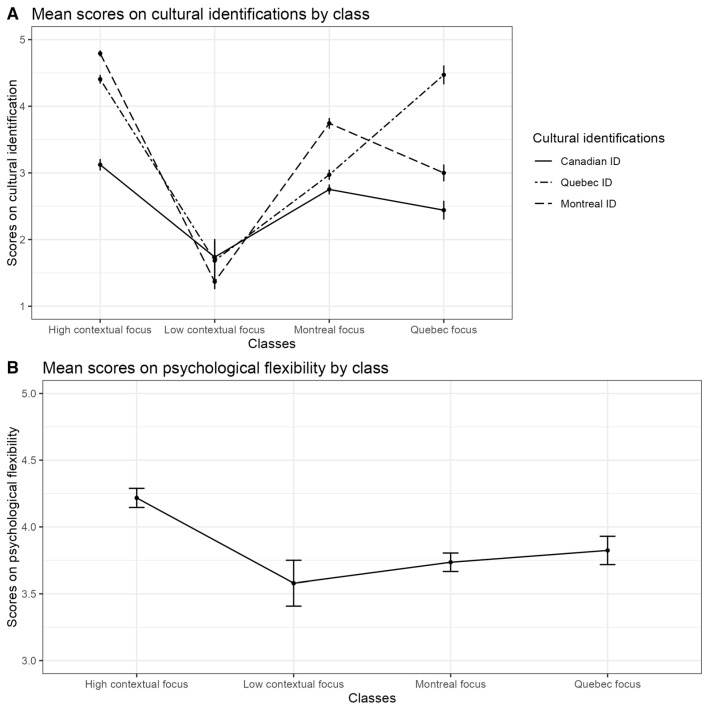

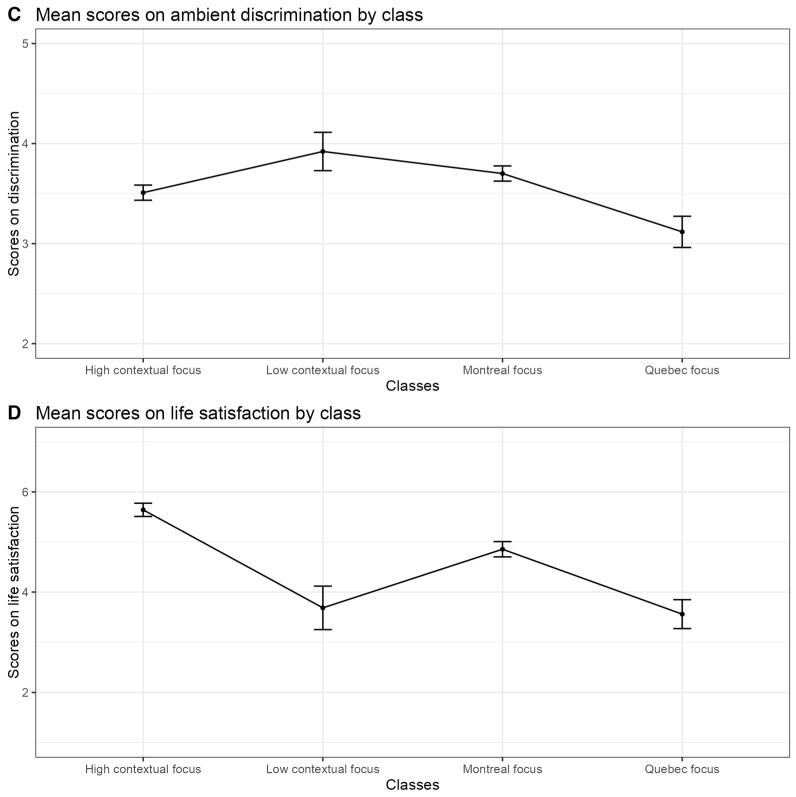
Mean scores on class belonging probability. *Note.* Error bars represent ±1 standard error of the mean.

Four identity classes emerged from the analysis (see Figure 1). The latent profiles identified in this study represent configurations of contextual identification with local (Montreal), regional (Quebec), and national (Canada) cultural reference frames. These identifications are treated as parallel and non-hierarchical, reflecting simultaneous engagement with multiple mainstream cultural contexts rather than nested or sequential identity levels. Accordingly, class labels are descriptive of patterns of contextual belonging and should not be interpreted as acculturation strategies in the traditional heritage–host sense, as heritage culture maintenance was not assessed.

The *High contextual identification* class (33.3%) reflected high identification with all three levels: Canada, Quebec, and Montreal. The *Montreal focus* class (44.6%) showed strongest identification with Montreal, moderate attachment to Quebec, and weaker ties to Canada. The *Quebec focus* class (13.8%) included participants who strongly identified with Quebec but had low identification with both Montreal and Canada. Finally, the *Low contextual identification* class (8.3%) showed low identification across all three cultural streams.

Although a high contextual simultaneous identification profile was present, it was not the most prevalent (contrary to H2). Notably, as shown in Figure 1, Pane A, Canadian identification was relatively weak across all classes, highlighting the stronger salience of local and regional attachments—likely because these are more embedded in participants’ everyday lived experience.

### 8.3. Predictors of Class Membership

We assessed the effects of predictors on probabilities of belonging to each class. Table 3 summarizes these results using log-odds coefficients, which indicate the strength and direction of the associations between the predictors and the odds of latent class membership.

#### 8.3.1. Montreal Focus Class

**Montreal Focus vs. High Contextual Identification Class**. The Montreal focus class exhibited unique predictors, supporting H1 that local-level cultural identification is a meaningful option for some individuals. Higher psychological flexibility reduced the probability of belonging to the Montreal focus class (*B* = −0.96, *SE* = 0.33, *p* < .01). Given that this profile reflects a narrower, context-specific identification pattern rather than a broad engagement with multiple cultural reference points, this finding is consistent with H3, which anticipated that higher flexibility would be linked to profiles involving broader or more complex multicultural identifications.

**Figure 2:**
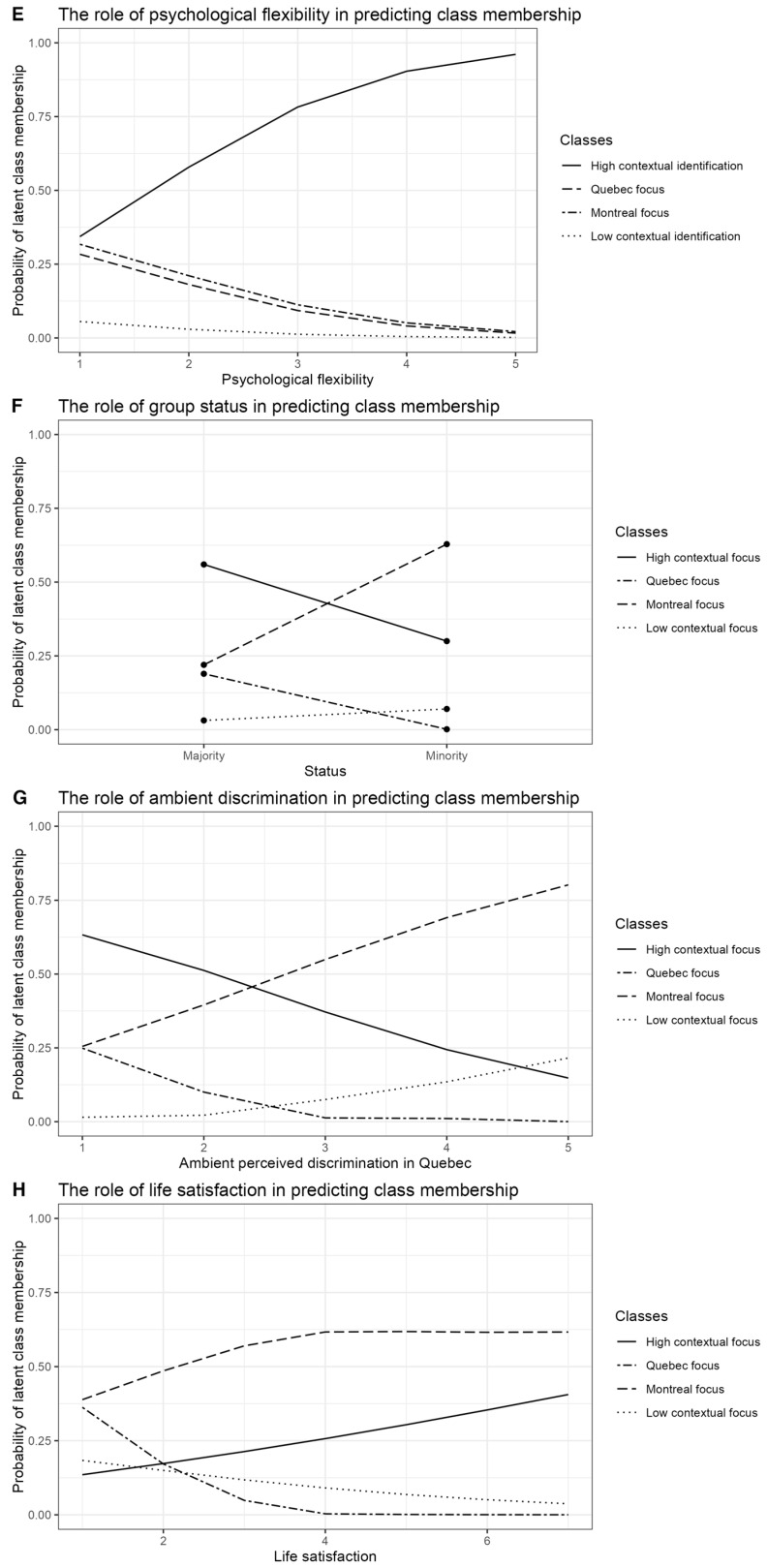
Covariates effects on class belonging probability. *Note.* Error bars represent ±1 standard error of the mean.

Older individuals were less likely to belong to the Montreal focus class compared to the High contextual identification class (*B* = −0.09, *SE* = 0.03, *p* = .001), with a 0.91 times lower likelihood. Minority group members were almost five times more likely to belong to the Montreal focus class compared to the High contextual identification class (*B* = 1.59, *SE* = 0.59, *p* < .01). This class was the only one where minority members had a higher likelihood of belonging. Pane F of Figure 2 illustrates this effect, showing overlap with High contextual identification but emphasizing local identification. Higher levels of perceived ambient discrimination increased the likelihood of belonging to the Montreal focus class by 2.13 times (*B* = 0.75, *SE* = 0.32, *p* = .02).

Pane F of Figure 2 illustrates this effect of group status on class belonging probability: Consistent with previous findings that minority members prefer integration among other acculturation strategies (Berry, 1997, 2003; Van De Vijver et al., 1999; Van Oudenhoven et al., 1998; Yu & Wang, 2011), minority status increased chances to belong to High contextual identification class over Quebec focus or Low contextual identification class. However, chances to belong to Montreal focus class for the minority members greatly overlaid those of belonging to High contextual identification class, suggesting the importance of local identification. The chances to belong to this class were also higher for those who perceived more ambient discrimination in Quebec (2.13 times more chances).

**Montreal focus vs. Quebec focus Class**. Striking contrasts emerged between the Montreal focus and Quebec focus classes. Minority individuals were 37.29 times more likely to belong to the Montreal focus class compared to the Quebec focus class (*B* = 3.62, *SE* = 1.25, *p* = .006). Older individuals were more likely to belong to the Quebec focus class (*B* = −0.09, *SE* = 0.03, *p* = .007). Higher life satisfaction increased the likelihood of belonging to the Montreal focus class by 2.56 times (*B* = 0.94, *SE* = 0.35, *p* = .01), while perceived ambient discrimination increased it by 5.13 times (*B* = 1.64, *SE* = 0.51, *p* = .002).

Perhaps the most important difference was observed between minority and majority members. Pane F of Figure 2 illustrates this effect, while Pane G reflects the effect of perceived ambient discrimination on the probability of belonging to the Montreal focus class compared to other classes. While more perceived ambient discrimination slightly increases chances to belong to Low contextual identification class, the probability to belong to either Integration or Quebec focus classes decreases. The most remarkable difference is observed for the probability of belonging to belong to High contextual identification class. At the same time, higher scores on ambient discrimination are associated with the highest probability of belonging to the Montreal focus class, suggesting a unique role of the city of Montreal in cultural identifications in the presence of ambient discrimination.

**Montreal Focus vs. Low contextual identification class**. No significant predictors differentiated the Montreal focus class from the Low contextual identification class.

#### 8.3.2. High Contextual Identification Class

**High Contextual Identification Class vs. Montreal Focus Class**. Minority members were significantly less likely to belong to the High contextual identification class than the Montreal focus class (*B* = −1.59, *SE* = 0.59, *p* = .01). Older individuals had higher odds of belonging to the High contextual identification class (*B* = 0.09, *SE* = 0.02, *p* = .001). Higher psychological flexibility increased the likelihood of belonging to the High contextual identification class (*B* = 0.96, *SE* = 0.33, *p* < .01) as demanding most complex cultural identification, supporting H3. Higher scores on perceived ambient discrimination decreased the likelihood of belonging to the High contextual identification class (*B* = −0.75, *SE* = 0.32, *p* = .02).

**High Contextual Identification Class vs. Quebec Focus Class**. Higher life satisfaction increased the odds of belonging to the High contextual identification class by 2.82 times (*B* = 1.04, *SE* = 0.31, *p* = .002). Perceived Ambient Discrimination: Higher discrimination levels increased the likelihood of belonging to the High contextual identification class by 2.41 times (*B* = 0.88, *SE* = 0.43, *p* = .04).

**High Contextual Identification Class vs. Low Contextual Identification Class**. Higher psychological flexibility was associated with 3.9 times greater odds of belonging to the High contextual identification class compared to the Low contextual identification class (*B* = 1.35, *SE* = 0.48, *p* = .007). Pane E of Figure 2 demonstrates this effect unequivocally. Increased perceived ambient discrimination was associated with a reduced likelihood of belonging to the High contextual identification class (*B* = −1.17, *SE* = 0.57, *p* = .044).

#### 8.3.3. Quebec Focus Class

**Quebec Focus vs. High contextual identification class**. Only two predictors were significantly associated with higher probability of belonging to this class: perceived discrimination and life satisfaction. Higher scores on ambient perceived discrimination in Quebec decreased chances to belong to this profile by 0.41 times (*B* = −0.88, *SE* = 0.43, *p* = .04), meaning that those who comprised this profile saw, on average, their province as less prone to discrimination. Interestingly, at the same time, these participants were less satisfied with their life in general, and those with higher scores on life satisfaction had 0.35 fewer chances to belong to Quebec focus class (*B* = −1.03, *SE* = 0.31, *p* = .002) than to Integration profile. Pane H of Figure 2 illustrates this effect: Higher scores on life satisfaction increase chances to belong to Montreal focus and High contextual identification classes, while chances to belong to Quebec focus class show a constant decrease, like the decrease in chances to belong to Low contextual identification class. In fact, as Pane D of Figure 1 shows, mean scores on life satisfaction in Quebec focus and Low contextual identification classes do not differ significantly (*t* = 1.44(39.61), *p* = .16).

**Quebec Focus vs. Montreal Focus Class**. Older individuals were more likely to belong to the Quebec focus class (*B* = 0.09, *SE* = 0.03, *p* = .006). Minority members, those with higher life satisfaction, and individuals perceiving higher discrimination were less likely to belong to the Quebec focus class compared to the Montreal focus class (*B* = −3.62, *SE* = 1.25, *p* = .006; *B* = −0.94, *SE* = 0.35, *p* = .01; *B* = −1.64, *SE* = 0.51, *p* = .002).

**Quebec Focus vs. Low Contextual Identification Class**. Belonging to a minority group and perceiving higher discrimination decreased the likelihood of being in the Quebec focus class compared to the Low contextual identification class (*B* = −4.15, *SE* = 1.66, *p* = .02; *B* = −2.05, *SE* = 0.69, *p* = .004). Table 3 summarizes these results.

## 9. Discussion

This study explored multicultural identity patterns across local (Montreal), regional (Quebec), and national (Canada) levels in a superdiverse urban context, using a person-centered approach. It also examined psychological flexibility as a predictor of identity complexity. We hypothesized (H1) that Montreal identification would emerge as distinct, (H2) that an integration profile would be prevalent, and (H3) that psychological flexibility would positively predict integration-like profiles. Broadly, our findings supported H1 and H3, though Montreal-focused identification proved more prevalent than full integration, offering important insights for contemporary models of cultural identity development.

### 9.1. Multicultural Identity and Place: Beyond National Frames

Our results confirmed that identification with Montreal was distinct from identification with Quebec or Canada, and often stronger, consistent with H1. This finding reinforces the proposition that local, place-based cultural identities serve as salient anchors, particularly in complex sociopolitical environments (Proshansky, 1978; Lewicka, 2011). Integration does not necessarily unfold solely between heritage and national mainstream cultures but may involve local level that embodies more inclusive and emotionally resonant possibilities of belonging relative to provincial or national mainstream identity frames, particularly in complex sociopolitical environments.

Montreal’s emergence as a distinct cultural identification possibility must be understood in light of its unique sociohistorical context. Positioned within a province that promotes interculturalism centered on French language and Quebecois traditions (Mathieu, 2022) yet situated in a federal country constitutionally committed to multiculturalism (Brosseau & Dewing, 2018), Montreal has developed a cultural ethos that often transcends, and at times resists, these broader frames. Its pluralistic character can resonate with both dominant and minoritized individuals who struggle to align their values or lived realities with the cultural expectations attached to Quebec or Canadian identity. For those who feel misrecognized or constrained by prevailing identity norms, Montreal’s multicultural fabric may offer a more inclusive and emotionally meaningful sense of belonging than that offered by provincial or national narratives, which may implicitly define “mainstream” in exclusionary ways (Alba & Duyvendak, 2020). This does not imply that local place-based identification supersedes ethnic, religious, or family identities, but rather that it may function as a comparatively accessible mainstream frame of belonging when provincial or national narratives are experienced as exclusionary.

The lower overall endorsement of Canadian identity across classes mirrors prior findings that national-level multicultural ideals do not always translate into lived inclusivity (Bilodeau & Turgeon, 2023). In Canada, cultural policies diverge across levels of governance—Quebec’s interculturalism versus Canada’s multiculturalism—which helps explain why identification with Quebec varied markedly across classes. Consistent with previous work (Berry & Hou, 2021), the *Quebec-focused* class showed Canadian identification levels nearly as low as those observed in the *Low contextual identification class*, with no significant difference between them (*t*(61) = 0.52, *p* = 0.603). Across nearly all other profiles, however, Quebec identification was consistently stronger than Canadian identification. Taken together, this pattern indicates that many participants may perceive Quebec and Canadian identities as distinct rather than nested. For some, interculturalism may reinforce strong provincial loyalty; for others, particularly minoritized individuals, it may function as a barrier, making provincial identification less accessible or desirable. The comparatively weaker resonance of national identification, especially among minorities, underscores that acculturation processes cannot be fully captured by a single “mainstream” category. Instead, they must reflect the layered, place-based, and relational nature of belonging in superdiverse societies, shaped simultaneously by inclusion and systemic exclusion.

Further, H2 predicted that a class reflecting strong identification with all three cultural streams would be the most prevalent, drawing on theories suggesting that integrative strategies tend to be psychologically advantageous and broadly endorsed (Amiot et al., 2018; Berry, 2010). While an High contextual identification class did emerge, characterized by high identification with Montreal, Quebec, and Canada, it was not the modal pattern. This offers two important insights. First, integration is clearly possible within a superdiverse context, but it may require reconciling heterogeneous and sometimes competing cultural narratives (e.g., Quebec’s interculturalism vs. Canada’s multiculturalism). Not all individuals appear equally motivated or equally positioned to perform this reconciliation. Second, the predominance of the Montreal-focused profile suggests that local integration may be more accessible or meaningful than multi-level integration extending to regional and national frameworks. Montreal, as a cultural milieu, offers a relatively inclusive symbolic landscape that can accommodate diverse identifications without demanding alignment with broader political or historical narratives. In contrast, provincial and national identities, particularly in Quebec, carry stronger normative expectations and may feel less permeable for both minority and majority individuals. More localized identification, such as Montreal identification, becomes an alternative to people from both majority and minority groups who otherwise find it difficult to reconcile their values with those associated with Quebec or Canadian identities. Thus, H2 is supported insofar as a high contextual identification profile exists, but the lack of prevalence of this class signals that full multi-level integration may not be necessarily the preferred or most feasible strategy in superdiverse settings.

### 9.2. Psychological Flexibility: A Catalyst for Identity Complexity

Psychological flexibility emerged as a robust predictor of belonging to the High contextual identification class, supporting H3. Controlling for life satisfaction and psychological health, individuals higher in flexibility were more likely to maintain complex, multilayered cultural identities encompassing local, regional, and national cultural streams. This finding suggests that flexibility plays an important role in enabling individuals to hold together rather than hierarchize or oppose multiple cultural meanings when constructing a multicultural identity. In this sense, flexibility functions as a psychological mechanism that supports identity complexity: The capacity to hold multiple representations, integrate divergent cultural frames, and navigate emotional dissonance without identity fragmentation (Hayes et al., 1999; Kashdan et al., 2020).

Importantly, this result expands conceptualizations of flexibility beyond the idea that it merely results from multicultural exposure. Much existing research treats flexibility as an outcome of engaging with cultural diversity—an acquired openness whereby repeated intercultural contact fosters cognitive broadening, reduced stereotyping, and more integrative thinking (Benet-Martínez et al., 2006; Crisp & Turner, 2011). Our findings complement this perspective but also underscore flexibility’s potential role as an antecedent of multicultural identity construction. Individuals who are more flexible may find it easier to tolerate ambiguity, navigate contradictory cultural norms, and maintain a coherent sense of self in settings where cultural expectations are heterogeneous or contested. This capacity is likely especially relevant in superdiverse contexts such as Montreal, where local, provincial, and national cultural frames coexist but do not necessarily align.

Recognizing this dual role raises important conceptual and methodological considerations. If flexibility both facilitates and results from engagement with cultural plurality, then the association between flexibility and integration may be reciprocal. More flexible individuals may be more willing or able to participate in diverse cultural environments, and such participation may further cultivate flexibility—creating a mutually reinforcing dynamic rather than a unidirectional causal pathway. From a methodological standpoint, a cross-sectional design cannot disentangle these bidirectional effects. Longitudinal or intervention-based research would be needed to determine whether psychological flexibility primarily acts as a precursor of identity complexity, a consequence of multicultural engagement, or part of a positive feedback loop linking the two.

Finally, the role of flexibility extended across identity configurations but did not uniformly predict all profiles. Flexibility distinguished the High contextual identification class from both the Montreal-focused and Low contextual identification classes, indicating that complex identity integration may require not only emotional openness but also a capacity to manage the competing symbolic demands embedded in Quebec and Canadian identities. That psychological flexibility did not predict membership in the Quebec-focused class is notable. This profile—marked by strong provincial attachment but comparatively low national identification—may reflect a more bounded, normatively anchored form of identification that does not require the negotiation of multiple, potentially conflicting cultural frames. In contrast, integrating Montreal, Quebec, and Canada simultaneously demands navigating plural and sometimes contradictory cultural narratives. Flexibility may therefore be especially relevant for profiles that require broader identity permeability and multi-level reconciliation, while playing a less central role in profiles defined by narrower or more exclusive symbolic boundaries. Taken together, these findings position psychological flexibility as an important resource for constructing complex multicultural identities, particularly in superdiverse contexts where cultural reference systems are layered, shifting, and not always mutually compatible. Flexibility emerges not only as an individual coping asset but also as a facilitator of more inclusive civic identifications, making it a promising target for future interventions aimed at enhancing adaptive identity development across both minoritized and dominant group members.

### 9.3. Contextual Role of Covariates

While psychological flexibility was the primary predictor of interest, some covariates included for adjustment purposes, most notably minority status, were associated with very large odds ratios and wide confidence intervals. This pattern likely reflects sparse cell counts and quasi-complete separation across certain class-by-predictor combinations, a known issue in multinomial latent class regression when class sizes are uneven. As a result, these estimates should be interpreted cautiously as indicative of direction and association rather than precise effect size. Accordingly, we frame these findings as descriptive associations rather than stable or generalizable estimates.

Age, life satisfaction, and ambient discrimination were included as contextual covariates to clarify the associations involving psychological flexibility and identity profiles. Their effects provide background conditions rather than explanatory mechanisms. As such, these associations should be interpreted descriptively, reflecting correlational patterns rather than directional effects. Higher perceived ambient discrimination was associated with reduced likelihood of multi-level integration and increased likelihood of belonging to the Montreal-focus profile. In contrast, higher life satisfaction tended to characterize the Integration and Montreal-focus profiles rather than the Quebec-focus profile. Age showed modest associations, with older individuals more likely to belong to broader (*High contextual identification* or *Quebec-focused*) configurations. These patterns suggest that the broader social climate and individual well-being may subtly shape how cultural identifications are configured, though they fall outside the study’s primary theoretical aims. Importantly, because ambient discrimination reflects perceived characteristics of the broader social environment rather than personal experiences of victimization, its associations with identification profiles should be interpreted as contextual signals of inclusion or exclusion rather than direct psychological effects on individuals. From minority stress and contextual identity perspectives, the implications of perceived discrimination climates are likely contingent on individual resources such as regulatory capacity or social support; however, examining such moderation effects was beyond the scope of the present study.

## 10. Theoretical and Practical Implications

These findings point to the need for multilevel models of cultural identity that move beyond the traditional national–heritage binary. In superdiverse cities, local identifications can function as primary anchors of belonging, particularly when provincial or national narratives feel normatively restrictive or exclusionary. The present results underscore that multicultural identity cannot be adequately understood without attending to contextual layers—local, regional, and national—that may function independently or in tension with one another. They also highlight psychological flexibility as a key intrapersonal resource for sustaining complex identity configurations, especially in environments where cultural expectations intersect and sometimes contradict.

Because psychological flexibility is malleable and responsive to intervention (Ditton et al., 2022; Peterson et al., 2024), this work also carries practical implications. Flexibility-enhancing interventions rooted in acceptance-based skills, perspective-taking, and value-directed action may support individuals in navigating cultural contradictions without identity fragmentation. For minoritized individuals, such interventions may strengthen coherence and resilience under conditions of systemic exclusion; for dominant-group members, they may broaden the symbolic boundaries of mainstream identity and reduce gatekeeping tendencies, thereby facilitating more inclusive relational and civic environments. Prior work showing that exposure to counter-stereotypic information can increase cognitive flexibility (Gocłowska et al., 2013) suggests one pathway through which such change may occur.

Concrete intervention strategies could be derived along two complementary lines. First, individual-level interventions such as brief psychological flexibility training modules, exercises that normalize cultural ambiguity, or structured reflection tasks encouraging individuals to articulate multiple values and identity commitments could help individuals integrate diverse cultural frames with less distress. Second, community-level interventions such as facilitated intercultural dialogue groups, workshops using counter-stereotypic exemplars, or local policy initiatives that explicitly frame municipal belonging as inclusive of diverse cultural histories could leverage psychological flexibility principles to widen shared narratives of belonging. While these strategies remain to be empirically tested, they represent feasible applications of the present findings and offer a roadmap for future intervention-oriented research.

## 11. Constraints on Generality

Several factors constrain the generalizability of our findings. The participant sample was drawn from adults living in Montreal, Quebec—a superdiverse, bilingual city with unique regional politics distinguishing between Canadian multiculturalism and Quebecois interculturalism. Results may not extend to rural populations, less-educated groups, or contexts where cultural diversity and nationalism are configured differently. Our operationalization of majority/minority status also reflects Quebec-specific racial and ethnic categorizations. Further, although our street-recruited sample reflects Montreal’s population distribution remarkably well, this very strength limits the interpretation of group-specific processes. Because the study was not designed to oversample minority or majority individuals, the results cannot speak definitively to the acculturation dynamics of either group in isolation. Consequently, this design offers a valuable population-level snapshot but restricts the depth with which minority acculturation and majority acculturation can be examined.

Second, the identity dimensions assessed—Montreal, Quebec, and Canada—are embedded in the specific sociohistorical and political context of Quebec. Because nonresponse to Canadian identification may reflect meaningful disengagement in the Quebec context, conclusions involving Canadian identification should be interpreted cautiously. In other regions, identity salience may be structured differently, requiring context-sensitive operationalizations. Thus, while the presence of multiple overlapping identities may replicate elsewhere, the specific content and hierarchy of identifications may vary.

Procedural aspects such as self-selected street recruitment and online survey completion may limit generalizability to individuals more comfortable with digital participation and multicultural discourse. Nonetheless, we expect the broad association between psychological flexibility and multicultural identification to be robust across recruitment methods targeting similarly urban and diverse populations. Also, the use of single-item measures for well-being and discrimination, while practical for reducing burden, may limit sensitivity. Future studies employing multi-item scales could refine these associations.

## 12. Limitations and Future Directions

Although this study advances a more nuanced understanding of multicultural identity development in superdiverse contexts, several conceptual and methodological limitations warrant consideration. First, the study operationalized multicultural identity through identification with three contextual cultural streams (local, provincial, national). This choice departs from process-based models (e.g., Amiot et al., 2007), which conceptualize multicultural identity as a developmental trajectory culminating in integrative synthesis. Our approach, while complementary, captures structural patterns of identification rather than motivational or developmental stages. Future research would benefit from integrating both perspectives to examine how structural profiles map onto longitudinal trajectories or identity motives. Also, the use of a general sample precluded direct modeling of heritage cultural identification, a dimension crucial for certain minority experiences. Future work should incorporate heritage dimensions where appropriate and explicitly model intersectional positionalities (e.g., race × language × migration history) to unpack possible structure of multicultural identity. Additionally, although the study was conducted in a superdiverse urban context, the operationalization of minority status relied on a binary distinction between individuals from the historically dominant group and those positioned outside it. This approach does not capture the full heterogeneity implied by superdiversity, such as differences between racialized and non-racialized minorities, linguistic minorities, or immigration generations, nor does it allow examination of how these dimensions may differentially shape identification processes. As a result, the observed associations between minority status, discrimination, and locally focused identification likely reflect multiple underlying mechanisms that cannot be disentangled in the present design. Importantly, the goal of this study was not to model diversity within minority groups, but to assess whether identification with a superdiverse metropolis emerges as a distinct component of the cultural self-concept. Future research with larger and more stratified samples is needed to examine how specific minority positions interact with place-based identity formation.

Further, psychological flexibility was measured as a general regulatory capacity rather than a culturally specific skill. This choice was intentional. Psychological flexibility, as conceptualized within ACT, reflects a foundational adaptive mechanism: maintaining perspective, tolerating internal conflict, and acting in accordance with values despite discomfort. Because acculturation is itself a form of adaptation, flexibility may operate as an underlying process that supports culturally situated decision-making without requiring a separate, domain-specific construct. Although developing a culturally tailored “intercultural flexibility” measure may appear appealing, it risks contributing to conceptual inflation by creating new constructs that simply repackage the same underlying mechanism and are validated by tools built to detect them. A more scientifically parsimonious approach is to investigate how existing constructs that appear adjacent (e.g., intercultural competence, bicultural identity integration) may share common regulatory processes rooted in psychological flexibility. Rather than multiplying terminology, future work should aim to clarify how these constructs converge on basic adaptive functions. This direction promises a more coherent account of multicultural identity construction that avoids fragmenting the field of social psychology already struggling with conceptual inflation (Mastroianni, 2023).

Several limitations qualify findings involving covariates. First, psychological well-being and life satisfaction could plausibly act as outcomes or mediators in identity processes rather than antecedent predictors; adjusting for them may therefore partially obscure indirect pathways. Given the cross-sectional design, the directionality of these associations cannot be established, and the present findings should therefore be interpreted as correlational. Longitudinal and experience-sampling designs are needed to disentangle whether well-being shapes identity configurations, emerges as a consequence of them, or both. Second, the use of single-item measures for well-being and discrimination, although efficient, limits precision and may underestimate associations. Finally, other unmeasured contextual factors (e.g., socioeconomic status, length of residence, neighbourhood dynamics) may also shape identity patterns; future research should incorporate a more comprehensive set of structural and psychological variables. Overall, these covariates help situate the main findings but do not alter the central conclusion: Psychological flexibility remains the most consistent predictor of complex, multi-layered identification patterns in a superdiverse urban context.

Further, complex identities are lived constructed processes that do not easily fit into preconceptualized measurements. Although the present study identifies statistical configurations of place-based identification, these profiles likely reflect distinct lived experiences shaped by structural, institutional, and interactional dynamics within the urban environment. For example, the Montreal-focused profile may emerge from everyday participation in a multilingual, cosmopolitan public sphere where local belonging is enacted through routine interactions in neighbourhoods, public transit, and workplaces, while provincial or national narratives remain symbolically distant or exclusionary. In contrast, the high context identification profile may reflect greater institutional alignment across levels of belonging, such as fluency in dominant languages, positive engagement with provincial institutions, and perceived legitimacy within national civic narratives, allowing individuals to sustain multiple identifications without pronounced tension. More Quebec-focused configurations may similarly reflect stronger alignment with provincial cultural norms and language regimes, reinforced through educational, media, or policy contexts that foreground Quebec as the primary locus of collective identity. Qualitative or mixed-method approaches could build on these interpretations by examining how daily interactions, institutional encounters, and symbolic representations of place translate into the identity configurations observed here, thereby linking statistical profiles to lived experiences of belonging, participation, and recognition.

Finally, data collection took place during the winter months, a period that may shape patterns of public-space engagement and affective connections to place in Montreal. However, recruitment was conducted primarily in highly trafficked public spaces, including metro station entrances, which remain central sites of daily activity across seasons. While this may reduce the impact of seasonal variation on sample composition, seasonal conditions could nevertheless influence how individuals experience and interpret local place-based belonging. Accordingly, the present findings should be interpreted as situated within a winter context, and future research should examine whether similar identity configurations emerge across different seasons.

## 13. Conclusions

This study highlights that multicultural identity in superdiverse contexts is not merely an additive sum of mainstream and heritage affiliations but a complex, multilayered process embedded in lived spaces of power, belonging, and exclusion. Montreal emerged as a salient cultural frame, offering a local belonging alternative for individuals navigating contested provincial and national narratives. Psychological flexibility proved crucial for sustaining complex, integrated cultural identities in this stratified environment. Ultimately, thriving in plural societies requires not only individual adaptability but collective re-imaginings of civic belonging. Psychological science must meet this complexity with models that honor the interplay of personal agency, systemic constraint, and the creative possibilities of multicultural identity.

## Figures and Tables

**Table 1:** Model Fit of Latent Class Analyses with Up to Four Latent Classes, N = 276.

	Number of Latent Classes			
	1	2	3	4
Maximum Log-Likelihood	−1163.57	−1097.76	−1057.26	−1027.43
No. of Estimated Parameters	12	32	52	72
AIC ^a^	2349.14	2255.53	2212.53	2190.86
BIC ^a^	2388.96	2364.14	2389.93	2437.04
CAIC ^a^	2399.96	2394.14	2438.92	2505.04
sBIC ^a^	2354.08	2269.01	2234.55	2221.43
Mean posterior probability	–	0.93	0.89	0.91
Relative Entropy	–	0.79	0.77	0.84
Bootstrap LRT ^a^, *p* Value ^b,c^	–	<.001	<.001	<.001

*Note.* ^a^ AIC, Akaike’s information criterion; BIC, Bayesian information criterion; CAIC, Consistent Akaike Information Criterion; sBIC, sample-size-adjusted Bayesian Information Criterion; LRT, likelihood ratio test. Incremental changes of BIC < 2 are considered marginal (Kass and Raftery (1995), p. 777). ^b^ Bootstrap LRT, Likelihood ratio test according to Nylund et al. (2007). ^c^ If <.05, will fit significantly better than a model with (k − 1) latent classes.

**Table 2:** Spearman correlations with confidence intervals.

Variable	*M* (*SD*)	1	2	3	4	5	6	7
1. Age	36.26 (14.67)							
2. Canadian ID	2.84 (0.94)	0.03						
		[−0.10, 0.16]						
3. Quebec ID	3.52 (1.26)	0.36 **	0.12					
		[0.25, 0.46]	[−0.01, 0.24]					
4. Montreal ID	3.89 (1.16)	0.14 *	0.30 **	0.40 **				
		[0.02, 0.25]	[0.18, 0.42]	[0.29, 0.49]				
5. PPFI	4.37 (0.74)	0.08	0.21 **	0.18 **	0.17 **			
		[−0.04, 0.20]	[0.08, 0.33]	[0.06, 0.29]	[0.06, 0.29]			
6. Well-being	3.18 (1.10)	0.26 **	0.05	0.20 **	0.12 *	0.33 **		
		[0.14, 0.36]	[−0.08, 0.18]	[0.08, 0.31]	[0.00, 0.23]	[0.22, 0.43]		
7. Life satisfaction	4.92 (1.78)	0.01	0.12	0.08	0.24 **	0.20 **	0.57 **	
		[−0.11, 0.12]	[−0.01, 0.24]	[−0.04, 0.20]	[0.12, 0.35]	[0.08, 0.31]	[0.49, 0.65]	
8. Ambient Discrimination	3.57 (0.85)	−0.1	−0.01	−0.24 **	−0.03	−0.02	−0.17 **	−0.01
		[−0.22, 0.02]	[−0.14, 0.13]	[−0.35, −0.12]	[−0.15, 0.09]	[−0.14, 0.10]	[−0.28, −0.05]	[−0.13, 0.11]

*Note.* Values in square brackets indicate the 95% confidence interval for each correlation. The confidence interval is a plausible range of population correlations that could have caused the sample correlation (Cumming, 2014). * *p* < .05. ** *p* < .01.

**Table 3:** Unstandardized coefficients for cultural contextual identification class membership.

	Montreal Focus vs.						High Context Identification vs.						Quebec Focus vs.					
	High Context Identification		Quebec Focus		Low Context Identification		Montreal Focus		Quebec Focus		Low Context Identification		High Context Identification		Montreal Focus		Low Context Identification	
Covariates	β [95% CI]	*p*	β [95% CI]	*p*	β [95% CI]	*p*	β [95% CI]	*p*	β [95% CI]	*p*	β [95% CI]	*p*	β [95% CI]	*p*	β [95% CI]	*p*	β [95% CI]	*p*
Psychological flexibility	**−0.97** [−1.22, −0.72]	.006	−0.15 [−1.36, 1.06]	.84	0.39 [−0.94, 1.72]	.39	0.96 [0.29, 1.63]	.006	0.82 [−0.89, 2.53]	.21	1.35 [0.09, 2.61]	.005	−0.82 [−2.09, 0.45]	.21	0.14 [−1.61, 1.89]	.84	0.53 [−1.10, 2.16]	.50
Age, years	**−0.09** [−0.13, −0.05]	.001	−0.09 [−0.15, −0.03]	.007	0.00 [−0.10, 0.10]	.93	−2.41 [−2.47, −2.35]	.001	0.00 [−0.04, 0.04]	.85	0.10 [−0.02, 0.22]	.07	−0.01 [−0.05, 0.03]	.85	0.09 [0.03, 0.15]	.007	0.10 [−0.02, 0.22]	.09
Minority status	**1.59** [0.02, 3.16]	.007	3.62 [−87.91, 95.15]	.006	−0.56 [−1.81, 0.69]	.62	−1.61 [−2.10, −1.12]	.007	2.03 [−0.51, 4.57]	.07	−2.12 [−4.86, 0.62]	.08	−2.04 [−4.86, 0.78]	.07	−3.51 [−4.68, −2.34]	.006	−4.61 [−6.03, −3.19]	.02
Gender male	0.63 [−0.43, 1.69]	.22	0.83 [−2.60, 4.26]	.28	−0.29 [−1.33, 0.75]	.69	−0.63 [−1.16, −0.10]	.22	0.20 [−1.07, 1.47]	.76	−0.92 [−2.21, 0.37]	.27	−0.20 [−1.07, 0.67]	.76	−0.83 [−1.90, 0.24]	.28	−1.11 [−2.62, 0.40]	.28
Well-being	0.12 [−0.57, 0.81]	.69	−0.38 [−1.05, 0.29]	.44	0.08 [−0.61, 0.77]	.80	−0.13 [−0.67, 0.41]	.69	−0.51 [−1.45, 0.43]	.24	−0.05 [−0.78, 0.68]	.90	0.51 [−0.42, 1.44]	.24	0.39 [−0.60, 1.38]	.44	0.46 [−0.27, 1.19]	.41
Life satisfaction	−0.09 [−0.46, 0.28]	.64	0.94 [0.21, 1.67]	.007	0.26 [−0.29, 0.81]	.21	0.10 [−0.33, 0.53]	.64	1.04 [0.31, 1.77]	.002	0.36 [−0.42, 1.14]	.19	−1.05 [−1.71, −0.39]	.001	−0.94 [−1.60, −0.28]	.007	−0.67 [−1.46, 0.12]	.10
Ambient discrimination in Quebec	**0.76** [0.01, 1.51]	.02	1.64 [0.38, 2.90]	.002	−0.43 [−1.06, 0.20]	0.39	−0.76 [−1.25, −0.27]	.02	0.88 [0.02, 1.74]	.04	−1.17 [−2.41, −0.07]	.04	−0.89 [−1.60, −0.18]	.04	−1.66 [−2.60, −0.72]	.002	−2.12 [−3.26, −0.98]	.004

*Note*. Values are log-odds coefficients (β) with 95% confidence intervals from latent class regression that reflect pairwise log-odds contrasts between latent classes. Several estimates, particularly those involving minority status, exhibit wide confidence intervals, reflecting sparse cells and potential quasi-complete separation; these associations should therefore be interpreted cautiously and as associative rather than causal.

## Data Availability

The raw data supporting the conclusions of this article will be made available by the authors on request.

## Supplementary Materials

The following supporting information can be downloaded at: https://www.mdpi.com/article/10.3390/bs16020195/s1, Table S1: Class Sizes (%) for Alternative Latent Class Solutions, Table S2: Mean Levels of Cultural Identification by Class for Two- and Three-Class Solutions, Table S3: Class solution treating cultural identification indicators as continuous, Table S4: Class-by-predictor contingency tables, Table S5: Ordinal comparisons of place-based identification (Wilcoxon signed-rank tests), Table S6: Complete cases robustness check.
