# Supplementary material for: Holding Complexity: Psychological Flexibility and Cultural Identity in a Superdiverse Urban Context
Source: Behav Sci (Basel). 2026 Jan 29;16(2):195. doi: 10.3390/bs16020195 (PMC12938506; doi:10.3390/bs16020195)
Supplement: Supplementary file 1 [file behavsci-16-00195-s001.zip › behavsci-4078904-supplementary.pdf]

Supplementary materials

Table S1. Class Sizes (%) for Alternative Latent Class Solutions

Model	Class 1	Class 2	Class 3
2-class	69.6%	30.4%	-
3-class	7.9%	48.5%	43.8%

Table S2. Mean Levels of Cultural Identification by Class for Two- and Three-Class Solutions

Identification	Class 1<br>M(SD)	Class 2<br>M(SD)
Canada	2.93(0.88)	2.47(0.96)
Quebec	4.11(0.93)	2.24(0.83)
Montreal	4.26(1.01)	3.08 (1.03)

Identification	Class 1<br>M(SD)	Class 2<br>M(SD)	Class 3<br>M(SD)
Canada	1.81(1.12)	2.97(0.91)	2.75(0.80)
Quebec	1.81(1.54)	4.48(0.63)	2.76(0.81)
Montreal	1.48(0.60)	4.37(0.91)	3.77(0.91)

Two-class solution

The two-class solution primarily distinguished participants based on overall level of cultural identification rather than qualitatively distinct configurations of belonging. One class showed relatively higher identification across all three levels (local, regional, and national), whereas the second class showed uniformly lower identification. Importantly, identification with Montreal, Quebec, and Canada varied in parallel within each class, indicating that this solution captured intensity of identification rather than structural differentiation among cultural reference frames.

Three-class solution

The three-class solution introduced an intermediate group between the higher and lower identification profiles, resulting in a gradient of identification strength (high, moderate, low). However, as in the two-class solution, the three identification domains largely covaried within classes. Local, regional, and national identifications were not empirically differentiated, and no class reflected a distinct place-based or context-specific pattern of belonging. Thus, while the three-class model increased granularity, it did not yield qualitatively new or theoretically meaningful identity configurations.

In contrast, the four-class solution differentiated identity profiles not only by level but by configuration, most notably isolating patterns in which local identification functioned independently from regional and national identification. This differentiation aligns with ecological perspectives on identity and with the study’s aim to capture place-based multicultural belonging in superdiverse contexts.

Table S3. Class solution treating cultural identification indicators as continuous

Profile (mclust, 4 components)	Quebec Identification M(SD)	Montreal Identification M(SD)	Canada Identification M(SD)	Class Size (n)	Class Size (%)
High contextual identification	0.84 (0.81)	1.11 (0.22)	0.1 (0.99)	100	36.23
Low contextual identification	−1.34 (0.65)	−0.81 (0.89)	−0.44 (1.12)	22	7.97
Montreal focus	0.41 (0.81)	0.95 (0)	0.24 (0.97)	117	42.39
Quebec focus	0.80 (0.73)	−0.06 (0.34)	−0.003 (0.86)	37	13.4

Note. Cultural identification indicators were standardized (z-scores) prior to analysis. Positive values indicate above-average identification relative to the sample mean; negative values indicate below-average identification.

A sensitivity analysis treating cultural identification indicators as continuous yielded a four-class solution with substantively similar profiles, including a locally focused class and an integration-like class, supporting the robustness of the primary findings. These classes were derived from a four-component Gaussian finite mixture model (VEI parameterization) estimated using the *mclust* package in R. Although information criteria favored a five-component solution, the four-profile model reproduced the core qualitative configurations observed in the primary latent class analysis, including concurrent high identification across contexts, a low-identification profile, a place-focused profile, and a province-focused profile. This convergence across modeling approaches supports the robustness of the substantive class structure to alternative measurement assumptions. Zero standard deviations reflect near-zero within-profile variance estimates for Montreal identification under the Gaussian mixture model and should be interpreted as indicating very low variability rather than literal absence of variance. This pattern reflects the strong discriminative role of local identification in profile formation.

Table S4. Class-by-predictor contingency tables

Class	Majority n(%)	Minority n(%)
High contextual identification	65 (0.61)	41 (0.39)
Quebec focus	33 (0.97)	1 (0.03)
Montreal	26 (0.22)	91 (0.78)
Low contextual identification	1 (0.21)	15 (0.79)

Ordinal robustness analyses.

Because identification ratings were assessed on ordinal 7-point scales, we conducted nonparametric robustness analyses to verify that conclusions did not depend on treating these variables as approximately continuous. Paired Wilcoxon signed-rank tests were used to compare identification with Montreal, Quebec, and Canada within participants. These analyses yielded the same pattern of results as the parametric tests reported in the main text.

Table S5. Ordinal comparisons of place-based identification (Wilcoxon signed-rank tests)

Comparison	V	p
Montreal vs. Quebec	11,030	< .001
Montreal vs. Canada	23,281	< .001
Quebec vs. Canada	16,000	< .001

Note. Tests are two-tailed Wilcoxon signed-rank tests with continuity correction. *V* denotes the Wilcoxon test statistic.

Table S6. Complete cases robustness check

Coefficients	Estimate	Std. Error	z value	Pr(>|z|)
Intercept	-6.882802	1.476242	-4.662	3.13e-06 ***
Age	-0.003964	0.011915	-0.333	0.73934
Discrimination	0.484804	0.234348	2.069	0.03857 *
Montreal identification	0.563507	0.205971	2.736	0.00622 **
Quebec identification	0.359049	0.178121	2.016	0.04383 *

Note. Signif. codes: 0 ‘***’ 0.001 ‘**’ 0.01 ‘*’ 0.05 ‘.’ 0.1 ‘ ’ 1
